# Potential communications: rethinking projective identification

**DOI:** 10.3389/fpsyg.2026.1770786

**Published:** 2026-01-30

**Authors:** Joona Taipale

**Affiliations:** Department of Social Sciences and Philosophy, University of Jyväskylä, Jyväskylä, Finland

**Keywords:** affective discharge, *as if*, communicative intention, communicative potential, crying, evacuation, expression, unconscious communication

## Abstract

The article focuses on the *communicative aspect* of projective identification. By distinguishing between “communicative potential” and “communicative intention” and applying this distinction to the theory of projective identification, the article argues that the communicative aspect of the latter can be interpreted in two different ways. As will be shown, the different interpretations suggest a remarkably different metapsychological picture of what is happening between the analyst and the patient. Given that the distinction between communicative potential and communicative intention has not been established in the available literature, the ongoing debate is thus haunted by a fundamental ambiguity, that has both theoretical and clinical consequences.

## Introduction

“It is a very remarkable thing that the *Ucs* of one human being can react upon that of another, without passing through the *Cs*. This deserves closer investigation, especially with a view to finding out whether preconscious activity can be excluded as playing a part in it; but, descriptively speaking, the fact is incontestable” (Freud, 1915, 193).

While contemporary psychoanalysis largely builds on the idea of *unconscious communication* between the patient and the analyst, the nature of this communication is still debated. Much has changed since the times of Freud and Klein, who both believed that the analyst’s affective involvement in the analytic process should be kept to a minimum. Freud famously compares the analyst to a surgeon who must “put aside all his feelings, even his human sympathy, and concentrate his mental forces on the single aim of performing the operation as skillfully as possible” (Freud, 1912, 115). Klein even argues that if the presence of the patient awakens strong emotional reactions in the analyst, the latter is not doing their work correctly (Spillius et al., 2011, 134). In contrast, contemporary clinical theories heavily emphasize the necessity of the analyst’s affective involvement. What Winnicott writes in the 1950s about the affective preoccupation of the devoted caregiver in the early stages of development is further developed by Bion in the 1960s into a theory of the analyst’s openness and affective investment in what they consider to be receiving from the patient. Bion believes that, like a mothering figure who adjusts to the needs and wants of a child who is still unable to articulate and represent these by themselves, the analyst cannot merely listen to the patient from a surgical distance but must also take in and “contain” the patient’s affective turmoil, allowing it to be articulated in the analyst in the guise of “daydreams” or “reveries,” thus enabling the patient to rediscover their turmoil in a more tolerable form.

In the present psychoanalytic scene, this *unconscious exchange* is termed “projective identification.” The term is an offspring of the Kleinian–Bionian tradition, but as a *general phenomenon* it is widely recognized across the different psychoanalytic “schools.” As Spillius and O’Shaughnessy put it, “projective identification gives a name to, and clarification of, the dynamics of direct communication and the phenomena of transference and countertransference that are universal to mankind” (Spillius and O’Shaughnessy, 2012, 366). Indeed, the phenomenon allows for various conceptualizations, and the abundance of available interpretations has led some scholars to doubt whether it is reasonable to encompass all of that under just one term (see Hinshelwood, 1991, 186–188).

The prevalent concept of projective identification is heavily influenced by Bion, and it can be divided into three components. First, there is the *phantasy element* of projecting part of oneself into the object. Second, there is the *manipulative element* of persuading the object to align with that phantasy, or striving to “induce the other to become the very embodiment of projection” (Laing, 1969, 111). Third, there is the *reinternalization element* of rediscovering in the object, in a more tolerable form, the part of oneself that has been projected (see Ogden, 1992; Sandler, 1987). For a standard example, consider an infant who is distressed, cries out, and thus reactively “evacuates” their plight—i.e., an unwanted part of themselves (Klein, 1946, 8)—into the caregiver, who is expected to digest their plight, as it were. Likewise, clinicians often report experiences where their patients seem to be giving them a “nudge” (Gabbard, 1995, 477) and “forcing stuff” into them (Winnicott, 1971, 181). During such “vexing moments,” to quote Seligman, the therapist “feels pressured or even coerced, consciously or unconsciously, to take on feelings or entire roles” that they do not recognize as their own (Seligman, 2018, 195; cf. Joseph, 1987, 73; Ogden, 1989, 26). Insofar as this affective intrusion makes the therapist feel what the patient is feeling, it can be considered a kind of *communication*. And insofar as the therapist is receptive and manages to “contain” what is thus “pushed into” them, the patient can, in turn, feel “understood” (Ogden, 1992, 21).

The present article focuses on this *communicative aspect* of projective identification and argues that it harbors a fundamental ambiguity with considerable theoretical and clinical consequences. To tentatively pinpoint the problem, note that the claim that the crying infant “makes the receiver feel their plight” can be understood in two ways, depending on whether one considers such “making” to be *intentional or unintentional*. On the one hand, one might think that the crying infant is *unconsciously trying to make* the caregiver feel their plight—either with them or for them. On the other hand, one might think that the crying infant *comes to make* the caregiver feel the plight, even if this was not their conscious or unconscious aim. In our terms, the first interpretation considers the affective pressure felt by the caregiver as a deliberate outcome of the infant’s unconscious “communicative intention,” whereas the second interpretation underlines the “communicative potential” of the cry and considers the felt pressure as something that can be used *as if* it harbored a communicative intention—and responded accordingly—without postulating one.

The distinction between “communicative intention” and “communicative potential” has not been previously established in the literature. This threatens the plausibility of the concept of projective identification itself and the theory building on it. From a clinical perspective, the distinction might first seem like an insignificant conceptual detail devoid of practical value: the clinician might find it useful to attend to the felt pressure *as if it harbored a communicative intention* no matter whether there *is* one. What is problematic, however, is when one slides from this “as if” attitude into metapsychologically postulating a communicative intention in the patient. The point is quite crucial. Depending on whether one assumes a communicative intention, one ends up with two significantly different metapsychological pictures of what is going on in the analytic situation—and this is destined to carry over to clinical work as well. My aim here is to offer an interpretative tool—not to dictate how things are. That is to say, instead of trying to challenge the widely documented *experiences* of the clinicians (and caregivers), I will offer a conceptual distinction that may be of use when *articulating and theoretically developing* such experiences. Yet, given that the mentioned ambiguity widely permeates the contemporary discussion of projective identification, unveiling and clarifying it may be expected to have a significant theoretical and clinical impact.

In what follows, (1) I will first show how the communicative aspect has found its way into the notion of projective identification and, against this background, (2) introduce the distinction between communicative intention and communicative potential. This enables me to (3) examine the sense in which affective discharge can be used as a mode of communication, (4) revise the nature of the projective phantasy that allegedly grounds the act of projective identification, and (5) elaborate on the consequences of my argument.

## 1 Projective identification as a mode of communication

While the term “projective identification” had already been used before her work, it was Melanie Klein who made it famous (see Klein, 1946; Spillius, 2012, 3–4). This is somewhat ironic, given that Klein herself never really liked the term and constantly referred to her famous 1946 paper, the second edition of which first mentioned the notion, as her “splitting paper” instead of her “projective identification paper” (Spillius, 2012, 6). Indeed, it was not Klein but her followers who systematically positioned the concept at the center of psychoanalytic theory and practice.

For Klein herself, projective identification is a combination of the mechanisms of *splitting*, *projection*, and *identification*, by which the individual organizes their experiential reality in the schizo-paranoid position (Klein, 1946, 4; Spillius et al., 2011, 131; Sandler, 1987, 13). Klein follows Freud in assuming that the functioning of the early ego is comprehensively governed by the pleasure principle. In effect, the self/non-self distinction is primarily determined by the affective quality of the respective content (see Freud, 1930, 67–68). In Kleinian terms, anxiety, distress, and other intolerable “parts” of the self are spontaneously *discharged* and thus quite literally *expressed*. To ward off their plight, the immature infant defensively attempts to *push it out* and *create distance from it*—much like we instinctually do with any unpleasant *external* stimuli, such as shutting our eyes and turning our head when there is a bright light—with the alleged aim of psychically “casting out” what is actually felt as part of the self.^1^ At the outset of development, Klein argues, “splitting, denial, and omnipotence” thus “play a role similar to that of repression at a later stage of ego-development” (Klein, 1946, 6; Bion, 1957b). As Klein adds, “*in so far as the mother comes to contain* the [projected] bad parts of the [infant’s] self, she is not felt to be a separate individual but is felt to be the bad self” (Klein, 1946, 8). In Winnicott’s terms, the mother then acts as a *mirror* in which the child sees themself (Winnicott, 1967, 152). By being split off and cast out in phantasy, the intolerable feeling does not truly lose its subjective quality—after all, “we must, in some way, be aware *that what we have projected is our own in order to feel the relief of being rid of it*” (Sandler, 1987, 26). Yet, the projective procedure allows *a certain distance* from the intolerable feeling: what is altered is “the *placing*” of the feeling (Freud, 1892, 208), which now seems to belong to the object. In phantasy, the infant thus “deposits” part of itself into the object, whereby one’s *own feeling* now appears *over there*—and this is not that far from saying that, in phantasy, the child finds the object to be feeling *their* feeling.

For our purposes, it is important to underline that Klein herself does not assign a communicative function to projective identification (see Spillius et al., 2011, 134). She considers it solely in terms of an *intrapsychic phantasy*. Indeed, she does note that “the effect of this phantasy is a very real one” (25), but with such “effects” she refers to changes in the projector’s intrapsychic landscape and conduct—not to changes in the *recipient* of projective identification. For Klein, projective identification is to be *interpreted* by the analyst from a professional distance—in her unpublished papers, as already noted, Klein straightforwardly emphasizes that the analyst cannot do their job if they allow the patient’s projective identification to invoke strong affects in them (see Spillius et al., 2011, 134). For Klein, Sandler summarizes, “projective identification was a process that occurs in phantasy. […] The real object employed in the process of projective identification is not regarded as being affected—the parts of the self put into the object are put into the phantasy object, the ‘internal object’, not the external object. […] Countertransference is scarcely mentioned, and when it is […] it is regarded as a hindrance to the analyst’s technique” (Sandler, 1987, 17).

In this regard, a significant landmark in the post-Kleinian evolution of the notion lies in Paula Heimann’s work on *countertransference*. In sharp contrast to Klein, Heimann suggests that the “analyst’s emotional response to the patient within the analytic situation represents one of the most important tools for his work,” and that the “analyst’s counter-transference is an instrument of research into the patient’s unconscious” (Heimann, 1950, 74; cf. Sandler, 1987, 17). The idea is that since the attitudes and behaviors of the patient toward the analyst are saturated and thus partly determined by their unconscious projective phantasies, the analyst’s countertransference arising in the presence of the patient is, in part, a reaction to—and hence a kind of window into—the patient’s unconscious phantasies. For instance, the analyst may feel that they are tacitly “suggested” a particular *role* by the patient’s conduct, as if the patient is “enlisting” the analyst “to enact with them scenes from their internal object world” (Ogden, 1992, 3; cf. 26). While the source of this “pressure” might be barely noticeable, and even if the analyst is destined to “enact” the role before realizing what has happened (Ogden, 1992, 43), the felt pressure, and the articulations that it spontaneously elicits in the mind of the analyst, can serve as a “very rich source of data about the patient’s internal world” (Ogden, 1992, 4). To quote Kernberg, countertransference figures as one of the three main sources of information in the analytic situation: “the patient’s subjective experience, communicated by means of free association; his nonverbal behavioral manifestations during the hour; and the analyst’s emotional response to these behaviors” (Kernberg, 1999, 172).

From acknowledging the clinical value of countertransference, there is a relatively short leap into the Bionian account. Extending Klein’s insight that the phantasy of projective identification comes with “real effects,” Bion considers projective identification as a concrete interactive process. By developing Heimann’s ideas of countertransference and elaborating upon how the pressure exerted by the patient awakens dream-like “reveries” in the mind of the analyst, Bion suggests that projective identification is “a method of communication” (Bion, 1957a, 99; see Lopez-Corvo, 2018, 62). What Bion has in mind is not communication in the everyday sense of the term, however. Rather than “informing the other” about something—which is a process mediated by symbols (e.g., words, gestures)—what we have here is communication in the sense of “having an impact on the other” (Hinshelwood, 2023b, 33)—an *immediate* or *direct* form of communication, whereby one individual “evokes” feelings in another (Ogden, 1992, 44; cf. Symington, 1985, 365).^2^ While this archaic and pre-symbolic mode of communication may be *emphasized* in deeply regressed or psychotic patients, in a less emphatic guise, it denotes an undercurrent in all human interaction (Sandler, 1987, 24; Bion, 1957c, 92; Bion, 1962, 37). In this sense, projective identification is a “normal” phenomenon (Bion, 1957a, 103): far from being confined to the consultation room, to varying degrees, the phenomenon is part and parcel of our everyday life (Sandler, 1987, 19; Ogden, 1994, 102; Rosenfeld, 2012, 80; Hinshelwood, 2018, 101, 201).

For a prototype of this archaic mode of communication, consider the cry of the infant. Rather than a symbolic message informing the caregiver *about* the state of the child, the cry *is* the child’s plight in concrete form: an unpleasant affect that the child spontaneously expresses or pushes out in an audible way. The caregiver receives this communication *as directly as one receives a cold shower*. For the caregiver, the plight of the child is a raw affective quality, a simple fact, and hence something that is immediately clear when hearing the cry; it involves no hidden meaning or repressed content that awaits psychoanalytic deciphering or interpretation. As Hinshelwood puts it, projective identification is “not the communication of information but an unsymbolized transfer of direct experiences” (Hinshelwood, 2023b, 44). This yet-to-be-contained and yet-to-be-symbolized affective quality is what the caregiver first and foremost *reacts to* and responds to, and they do this spontaneously, by engaging in “imaginative elaboration” (Winnicott, 1949, 244) of the raw affective pressure they feel. This impact, in turn, changes the way that the caregiver dwells in the situation, so that the presence of the sensitive caregiver comes to provide, for the infant, a way to *contain* the hitherto uncontained and hence non-represented affect of the infant.

The same applies, *mutatis mutandis*, to clinical encounters. The analyst is expected to “receive” (Ogden, 1992, 22) the affective pressure exerted by the patient, convey a nascent non-verbal articulation of it, and thus *give shape and form* to their raw affects in the guise of “dreams” (Ogden, 2022a, 99; cf. Brown, 2011, 208), “reveries” (Bion, 1962, 36), “visual images” (Busch, 2019, 34), “hallucinatory perceptions” and other kinds of “figurations” (Botella and Botella, 2005, 10). Much like the caregiver’s presence gradually enables the child to mentalize their feelings, the analyst is asked to “metabolize” the patient’s raw affects and thus contain the uncontained affect *for them*. Such analytic receptivity enables the patient to reinternalize what they had “evacuated” into the analyst (Bion, 1962, 59), for, as Ogden puts it, in projective identification the “patient learns from what was his to begin with” (Ogden, 1992, 40).

The difference from Klein is thus clear. While Klein views projective identification as a *phantasy* where the object assumes the role or feeling that one is trying to get rid of (Spillius et al., 2011, 126; Spillius, 2012, 6), Bion adds that the projective phantasy is accompanied by an *actual pressure* that manipulates the object to *actually* “live out” the projected role and thus *actually* take in what is “pushed” into them (e.g., Bion, 1961, 213; Joseph, 1987, 73). Departing from Klein, Bion considers the analyst’s use of their own emotional responses and countertransference feelings as one of the basic tools of psychoanalysis. While admitting on the one hand that this “use of counter-transference” is “an expedient to which we must resort until something better presents itself” (Bion, 1955, 78), he also notes that sometimes projective identification is “the only method of communication by which [the patient] feels he can make himself understood” (Bion, 1957a, 105).

Naturally, what complicates matters is that, as an undercurrent in all human interaction, projective identification equally characterizes *the analyst’s relation to the patient*. In other words, the analytic setting is a *reciprocal field* where the analyst and the patient unconsciously react and respond to each other, hence metabolizing, mentalizing, and symbolizing each other’s affective experiences within the limits of their own abilities. As Ogden puts it, “the creation of an analytic process depends upon the capacity of analyst and analysand to engage in a dialectical interplay of states of ‘reverie’” (Ogden, 1996, 883). And as projection and identification occur simultaneously in both members of the interaction, this largely unconscious process of interchange is given “immense complexity and variability” (Spillius, 2012, 60).

## 2 Communicative intention and communicative potential

Looking at the evolution of psychoanalytic thinking from Klein through Heimann to Bion, we can witness a shift of emphasis from *the mind of the patient* to *the mind of the analyst* (see Reis, 2021). It is worth underlining that it is *in and through this shift* that projective identification is “discovered” as a mode of communication. There are two ways to interpret the nature of this “discovery,” however, and likewise two ways to interpret the “discovery” that an individual analyst makes when grasping the pressure they feel as an unconscious communication from the patient. On the one hand, one might assume that the mentioned “discovery” is simply a matter of *unveiling* an unconscious attempt to communicate that characterized the pressure all along. On the other hand, one might think that the “discovery” involves *using* the felt pressure as if it was the patient’s attempt to communicate something and hence *establishing* it as such.

To illustrate what is at issue here, let me give an example. Consider someone in the room looking miserable, breathing heavily, and sneezing. Whether you are an analyst or not, the other’s bodily presence makes you aware that they are feeling “under the weather.” Importantly, you become aware of the other’s feeling state not only by inferring from their external appearance but also from the way their presence makes you feel—owing to the mirror resonance system of the brain (see Schoenewolf, 1990, 58; Gallese, 2009, 531; Arizmendi, 2011, 406), the other’s perceived malady is spontaneously echoed or “simulated” in your own body (Sandler and Joffe, 1967, 219). Now, you can act *as if* the other’s sneeze was their way of *telling you* that they feel ill (*as if* it was an intentional communication) and respond accordingly: you might verbally say, “I hope you feel better soon,” to which the other person responds with a thanks. However, the fact that you can successfully *use* the other’s sneeze as a communication does not imply that they were *trying to* communicate their malady to you. The difference in your presumed conduct would be significant if you assumed that the other’s sneezing was their unconscious attempt to inform you about their malady, craving your attention or sympathy, or even manipulating you into feeling their feelings. In both cases, the other’s bodily feeling can be said to be “pushed into” you, but it makes a huge difference whether you view the situation as a *deliberate attempt* to make you feel something or as something that you *use* for communicative purposes.

In our terms, the difference is between a “communicative potential” and a “communicative intention.” The point I want to make is that the former does not necessarily imply or presuppose the latter. I define “communicative intention” as a *conscious or unconscious attempt to make someone feel or experience something*. With “communicative potential,” in turn, I refer to something that can be *used* as communication and treated *as if it harbored* a communicative intention, yet without taking a stand on whether it *actually harbors* such an intention. In terms of the previous example, the other’s sneeze has a communicative potential, even if it might not harbor a communicative intention. In other words, you can successfully treat the other’s sneeze *as if* it were their way of “telling” you that they feel ill, and a communicative relationship may grow out of that, but to do so you do not need to assume that the sneeze actually was the other’s unconscious attempt to communicate something to you. As we can see here, the notions of communicative intention and communicative potential are not on equal footing: all acts involving a *communicative intention* arguably have a *communicative potential*, but not the other way around: for not all that can be used *as if* there was a communicative intention actually involves a communicative intention. The theoretical choice, therefore, is not between communicative potential and communicative intention, but more accurately on *whether to assume a communicative intention*.

This distinction outlines two different interpretations of the communicative aspect of projective identification. Yet, a challenge emerges here since, on the receiver’s end, projective identification may appear quite similarly *regardless of whether there is a communicative intention or not*. To underline this point and develop its consequences, we should take a closer look at the communicative potential of *affective discharge*.

## 3 The communicative potential of affective discharge

Of the three elements—the *phantasy element*, the *manipulative element*, and the *reintrojective element*—it is arguably the *manipulative element* that conveys projective identification with a communicative aspect. Certainly, the first element—namely, the unconscious phantasy of “abrogating” part of oneself and relocating it in the other (Ogden, 1994, 103)—does not provide projective identification with a communicative quality, no more than dreaming of a communication transforms that dream into a form of communication. The third element, again, corresponds to what in explicit communication counts as a *response to* or *an outcome of* communication. In other words, while the phantasy element allegedly *fuels* the archaic communication (see Kernberg, 1987, 101), and while reintrojecting what has been communicated allows the individual to *move forward* and thus benefit from the archaic communication (see Ogden, 1992, 12, 21), the manipulative element is the communicative *engine* linking the first and third elements.

In light of the distinction between communicative intention and communicative potential, the manipulative element allows for two different interpretations. *On the one hand*, the “pressure exerted” can be considered a set of behaviors that *aims* to make the object feel what one feels; it can be viewed in terms of an “unconscious intention to bring about a specific response in the analyst” (see Sandler, 1993, 1105). *On the other hand*, the “pressure exerted” can be viewed as a *byproduct* of affective discharge. In other words, while in the first interpretation, affective discharge is considered a *means* of influencing another individual, in the second interpretation, affective discharge is considered as a *goal in* itself. In terms introduced above, the first interpretation posits a “communicative intention” in affective discharge, whereas the second interpretation emphasizes the “communicative potential” of affective discharge and views the manipulative effect as one of its possible outcomes.

In psychoanalytic research literature, these two interpretations—and hence the two interpretations of the communicative aspect of projective identification—remain largely undifferentiated. To give one example, consider Ogden’s definition:

“Projective identification is a means by which the infant can feel understood *by making the mother feel* what her child is feeling. The infant cannot verbalize his feelings *so instead must induce those feelings in the mother*” (Ogden, 1992, 22; *my emphasis*).

In light of the distinction introduced here, the words in italics can be interpreted in two ways. On one hand, Ogden could be seen as siding with the first interpretation and suggesting that the infant is unconsciously *trying to make* the mother feel what they are feeling, thus *deliberately* inducing specific feelings in her. Yet, on the other hand, the same words could be taken to support the second interpretation and suggest that the affective presence of the infant *comes to* induce feelings in the caregiver or *comes to* make the mother feel something that the infant is feeling, because this is how affects operate: they are manipulative by nature, as it were. In our terms, the first interpretation suggests that the cry of the infant involves a “communicative intention,” whereas the latter suggests that it harbors a “communicative potential” that the caregiver can utilize and respond to, *as if* there was an intention. While both kinds of cases are undoubtedly found in clinical encounters, particularly with children of a certain age, conceptually distinguishing between the two scenarios seems highly important. After all, depending on which interpretation one embraces, one will entertain a very different metapsychological picture of the ongoing interaction.

To illustrate this further, consider the two cries of the child. Undoubtedly, what could be called the “spontaneous cry” of the newborn is full of *communicative potential*. The caregiver can treat it *as if* it was an intentional act of reaching out (e.g., “notice my plight!”), *as if* it was an explicit act of communication (e.g., “I am hungry!”), or *as if* it was a call for action (e.g., “feed me!”). That the *caregiver* tends to read communicative intentions into the cry, however, is by no means a guarantee that such intentions exist in the infant. Yet, intended or not, the affective discharge of the child influences the sensitive caregiver both externally and internally, and thus exerts an affective pressure upon the latter. The cry of an infant is one of the most affect-provoking sounds that exist; the plight of the infant is compelling and pressing for the recipient, and finding it “pushed” onto oneself is likely to give rise to strong emotional responses in the recipient (see Winnicott, 1947). Yet, none of this presupposes a communicative intention in the child. However, over time, the infant learns to use their cry to actively modify and thus intentionally alter the environment (Bion, 1962, 83). The ensuing “strategic cry” involves a *deliberate aim at influencing* the caregiver—even if this aim might remain unconscious. For sure, the distinction between the two cries is partly artificial, in that the strategic cry is not a new kind of cry altogether, but rather a spontaneous cry saturated by a certain degree of intent (cf. Bell and Ainsworth, 1972). Yet, it is crucially important to notice that this cry *builds on* the mechanism familiar from the initial “spontaneous cry,” as it is from the consequences of the latter that the child learns. In other words, provided with good-enough care, the infant increasingly becomes familiar with the “communicative potential” of their crying—i.e., they discover a pattern between the spontaneous cry and the sense of being cared for (Taipale, in press)—and this dawning realization enables them to tentatively *use* crying in a more or less deliberate or strategic fashion that merits the qualification of a “communicative intent.” For sure, if the needs of the child are not sufficiently met, after a while the child no longer cries: the communicative potential of the spontaneous cry is insufficiently realized, preventing it from being discovered by the child as something that can be intentionally used.

Freud underlines the ontogenetic primacy of affective discharge over communication: “[d]ischarge represents the primary function of the nervous system” (Freud, 1950, 296). According to Freud, the burdened psyche primarily seeks to offload its burden and thus restore homeostatic equilibrium—rather than paying attention to what the caregiver feels or does not feel. However, referring to the birth of what we call the “strategic cry,” Freud notes that when the child cries, “the attention of an experienced person is drawn to the child’s state by discharge along the path of internal change. In this way, this path of discharge acquires a secondary function of the highest importance, that of communication” (Freud, 1950, 318). To rephrase this in our terms: affective discharge (e.g., spontaneous crying) has a “communicative potential” that the caregiver can utilize, but this is not *why* the discharge initially occurs. That is to say, infants do not initially cry *in order to* be cared for; affective discharge does not initially harbor a communicative intention.

Here one might want to object in reference to the *evolutionary* significance of crying. Indeed, there is no denying that natural selection is more likely to have weeded out infants that do not cry when distressed, as they *therefore* fail to attract the caregiver’s attention when in need of nutrition, for instance. But natural selection only cares about *outcomes* and *survival*; it knows nothing of *meanings and intentions*. It is, of course, probable that infants who cry when distressed are more likely to receive care, survive, and have offspring, and this enables us to argue—*teleologically* and *après coup*—that crying “led to” communication and care, but it would be adultomorphic and incongruous to infer that *this was why the infant cried in the first place*. As should be clear by now, the communicative function is “secondary,” not in the sense of being less important, but in the sense of growing out of the primary function of affective discharge. The point to be emphasized, instead, is that the latter already involves a communicative potential, and sufficiently recognizing this potential is what enables the child to start using their cry more or less intentionally. Affective discharge has a “communicative potential,” and *in fortunate cases* it can influence those around the baby and thus attract their attention in an evolutionarily significant way, but to infer from this that affective discharge involves a “communicative intention” would be a teleological fallacy.

The distinction between the two interpretations of the manipulative element interestingly aligns with the distinction between the two types of cries. The spontaneous cry is an act of discharge and involves no communicative intention; however, it does possess communicative potential, as it can be interpreted by the receiver and responded to as if it was meant as a communication (such as a request) by the child. The strategic cry, in contrast, involves a communicative intention: it deliberately uses the communicative potential of crying to influence or manipulate the caregiver. As we have seen thus far, while both cases involve an affective pressure, there is a sequence of emergence between the two; the emergence of the strategic cry *presupposes* good-enough care, where the caregiver responds to the spontaneous cries of the infant. As a self-purposive expression of the child’s plight, spontaneous cry affects the recipient without a communicative intention, whereas the strategic cry is an other-directed expression with a communicative intention.

On the side of the recipient, however, the effect of these two cries may be very similar and even indistinguishable. The same applies to the analytic situation. Let me illustrate this with an imaginary example. On one hand, consider Patient 1, whose cognitive functioning is significantly lowered due to emotional stress and sleep deprivation over the past few weeks. This has affected their self-management, causing them to arrive late to analytic sessions, miss one meeting, feel lost, and express a sense of hopelessness vis-à-vis their therapeutic prospects. By contrast, consider Patient 2, who experiences a crushing feeling of worthlessness and entertains an unconscious fantasy in which their sense of worthlessness is projected onto the analyst. To induce such feelings in the analyst, the patient arrives late to sessions, misses a session, and expresses their sense of hopelessness and being beyond help in various ways. In both cases, we can easily imagine that the patient’s forgetfulness, tardiness, and feelings of being at a loss can lead the analyst to feel that their significance is downplayed, that their time is not appreciated, that they are unable to help the patient, and thus that they are worthless. Yet, whereas in one case the behavior of the sleep-deprived patient comes to make the analyst to feel worthless, in the other case such feelings stem from the patient’s unconscious *attempt to make* the analyst experience the patient’s sense of worthlessness. The million-dollar question is: *how can we distinguish which situation is the* case? In our terms, the problem is how to tell the difference between cases with a *communicative potential to be used* and cases with a *communicative intention to be revealed*. While clinicians are likely dealing with both kinds of cases, the difference between the two scenarios is highly important, as each is accompanied by a different set of theoretical commitments and clinical implications. It is always possible to *read* an intention where there is none, such as when interpreting a spontaneous cry as a disguised strategic cry, and the difficulty of distinguishing between these cases only adds to the importance of thinking about the distinction.

## 4 Projective phantasy revisited

Having introduced two possible interpretations of the *manipulative element* in projective identification, we turn to the *phantasy element*, namely, the phantasy that the individual is presumably trying to realize by exerting pressure. Along with the two possible ways to interpret the affective pressure, we are also left with two possible interpretations of the fueling phantasy. In the standard Kleinian–Bionian view, the phantasy is about pushing part of oneself *into the other*—whereby the patient’s conduct is taken as an unconscious attempt to realize *this* phantasy. By contrast, if the nature of the underlying phantasy is viewed as arising from the more elementary *need to discharge affective pressure*, we can assume that the phantasy is not primarily about pushing part of oneself *into the object*, but about pushing part of oneself *away*. Thus, in both models, we have a phantasy fueling the “exertion of pressure,” but whereas in one case the phantasy is about *getting rid of a feeling*, in the other case it is about *making someone else feel a feeling*.

Our considerations thus far have outlined two ways to interpret the communicative aspect of projective identification. In *the dominant Kleinian–Bionian view*, (1) there is the projective phantasy of relocating part of oneself *in the object*, (2) there is the attempt to make *this* phantasy true by exerting pressure upon the object, and if successful, this enables (3) re-identifying with what one had unconsciously deposited in the object (Ogden, 1994, 99). In what could be called *the discharge model*, as introduced above, the clinical building blocks of projective identification are present, but their metapsychological structure and phenomenology are understood differently. Here (1) the *phantasy element* is associated with the unconscious representation of the need to discharge the affective pressure, meaning that the phantasy is not about *pushing a feeling into someone else*, but about *pushing a feeling out*. And so, (2) the “manipulative element” can be viewed in terms of the inherently manipulative nature of affects and treated as a *byproduct* of affective discharge. Finally, (3) if the object is there to contain, mirror, or metabolize the affect that they end up feeling, this is perceivable both externally and internally by the individual, which means that (3) presence of the object comes to serve as an alternative (more digested, contained, and hence tolerable) expression of the feeling of the individual, enabling them to identify anew with the feeling they were trying to dispense with.

Again, my point is *not* to promote a choice between the two interpretations or suggest that the “discharge model” should be applied to all cases of projective identification—as stated, both interpretations have their place in clinical practice, and hybrid forms between them are always possible. My point is simply to make the distinction, which I consider crucial since the choice between them largely prefigures the scope of what the clinician will *look for* and hence also what they will *find*. As will be shown, these two interpretations are not sufficiently differentiated in the available literature. Taking a closer look at this enables us to assess the importance, as well as the theoretical and clinical implications, of the distinction between *communicative intention* and *communicative potential*.

While presenting projective identification as a mode of communication, Bion compares the uncontained “beta elements” with what Freud calls “accretions of stimuli” (Bion, 1962, 83; Freud, 1911, 221), and argues that *projective identification must be viewed as a functional extension of affective discharge*: “through projective identification thought itself takes on the function previously entrusted to motor discharge—namely ridding the psyche of accretions of stimuli” (Bion, 1962, 83). Echoing Freud’s distinction between the primary function of discharge and the secondary function of communication, Bion further specifies that projective identification *may be* or *may not be* “directed to altering the environment, depending on whether the personality is directed to *evasion* of frustration or *modification* of it” (Bion, 1962, 83). This distinction aligns with the two cries discussed above: while both influence the caregiver, the spontaneous cry aims at discharge, evacuation, or expression, whereas the strategic cry *additionally* involves an attempt to have an impact on the caregiver.

However, the distinction between modification and evacuation—and hence communication and discharge—is blurred in Bion’s theory of projective identification. On the one hand, Bion claims that projective identification is *something more than* evacuation; he emphasizes that it is not just the receiver but also the projector who assigns it a communicative function, and thus suggests that projective identification *involves a communicative intention*: “projective identification […] is felt by the patient to be a method of communication” (Bion, 1957a, 99). On the other hand, Bion *equates* projective identification with evacuation and discharge that *involves no communicative intention*: he associates projective identification with the “procedure for unburdening the psyche of accretions of stimuli” (Bion, 1962, 31), and underlines—like Freud—that “before the establishment of the reality principle, [crying] is not directed to alteration of the environment but to an unburdening of ‘the mental apparatus of accretions of stimuli’” (Bion, 1958, 79, 83).

Bion thus claims both that projective identification aims at modifying the environment (for example, influencing the caregiver or the analyst) and that it does not aim at modifying the environment but amounts to evacuation or discharge instead (without the intent to influence the caregiver or analyst). One passage that clearly reveals this uncertainty can be found in *Learning from Experience,* where Bion notes that an infant employing projective identification “has contact with reality sufficient to enable him to act in a way that engenders in the mother feelings that he does not want to have, or which he wants mother to have “(Bion, 1962, 31; Lopez-Corvo, 2018, 100). The “or” in the passage is quite significant. Certainly, the patient’s manner of being may engender feelings in the analyst, but whether this manipulative impact is driven by the phantasy of *warding off* intolerable feelings or by the phantasy of pushing intolerable feelings *into the object* results in two very different theories of what is happening between the two individuals. The attempt to push intolerable feelings *away* (i.e., anywhere but here) is different from the attempt to push intolerable feelings *into another experiencing being*. While both attempts can be used for communicative purposes *by the recipient*, Bion vacillates between considering projective identification in terms of *communicative intention* and in terms of *communicative potential*.

In Bion’s wake, the same ambiguity is widely reflected in contemporary discussions on projective identification. While communicative intentions are sometimes explicitly assigned to projective identification and sometimes not, most authors remain ambiguous about this. Let me provide three brief examples.

Elizabeth Spillius describes the manipulative aspect of projective identification by stating that the act is “unconsciously designed to evoke a specific response from the object” (Spillius, 2012, 58). As the phrasing makes clear, Spillius assumes that the bodily presence of the patient not only *happens to* evoke but is unconsciously *meant to* evoke a particular kind of response in the analyst. While the meaning of the phrase “unconsciously designed to” is unclear, I take Spillius to refer not just to the evolutionary function of the affective presence of the individual—for it would make no sense to say that the evolutionary function of affectivity is unconscious, just as it makes no sense to say that the evolutionary function of the umbilical cord is unconscious. Though she is not explicit about this, in our terms, Spillius seems to read a *communicative intention* into the theory of projective identification and correspondingly assume that the patient’s phantasy is not just about pushing something *out* but about pushing something *into the other*.

In a seemingly sharp contrast, Betty Joseph notes that “projective identification is, by its very nature, a kind of communication, even in cases where this is not its aim or intention” (Joseph, 1987, 67). Joseph supports her claim by stating that projective identification is, by definition, a process of “putting parts of the self into an object” (ibid). In other words, she justifies her assertion about the nature of what we call the *manipulative element* in reference to her assumption concerning the nature of the underlying *phantasy element* and thus posits a communicative intention. However, by presenting this *assumption* on the one hand and using it as a *justification* for the claim about the communicative nature of the act on the other, Joseph’s argument is circular. What it proves is a tautology: *insofar as* the phantasy fueling projective identification is about putting part of oneself into the other, the act of trying to fulfill *this* phantasy is a communicative attempt, since it *tries* to make the other feel something. Yet, instead of expressing any caveats, Joseph seems to treat her assumption as an unchallengeable truth and thus ends up already *presupposing* what she means to *explain*. On the other hand, having declared that the act is a form of communication “by its very nature”—and hence presumably whether it is received or not—Joseph adds that projective identification can be a mode of communication only if the analyst “tunes into” the patient and remains “open to” what is projected into them (Joseph, 1987, 71–73). In this manner, while asserting a communicative intention, Joseph nonetheless vacillates between the two interpretations we have outlined.

Robert Hinshelwood explicitly argues that projective identification unconsciously *aims at* “containment,” and as such, it is something more than an “evacuatory” act of “getting rid of fragments of the mind just to get rid of them, anywhere, without a thought of communication” (Hinshelwood, 2023a, 141). However, in the sentence that follows, Hinshelwood adds that the analyst’s task is to “lever” the expelled contents into “some restored representation that *can have* a communicative property” (ibid.; *my emphasis*). That is to say, Hinshelwood first suggests that projective identification is accompanied by something like a “thought of communication,” but then states that it is the receiver who assigns a communicative property to the felt pressure. In a circular manner, he ends up suggesting that the analysand’s receptivity to what the analyst gives back to them can be taken as an “empirical confirmation” of the fact that there *was* an unconscious communication all along (ibid.). In our terms, Hinshelwood first refers to a *communicative intention*, then shifts to speaking of a *communicative potentiality*, and finally justifies the former in reference to the latter—thus falling victim to the teleological fallacy that we articulated above.

What is at stake in all three examples is, in our terms, a conflation between “communicative intention” and “communicative potential”—all three *read in* a communicative intention but struggle to reconcile this assumption with the role of reception. As long as this distinction is omitted, the sense in which projective identification is a form of communication remains obscure. This haunts the theory in the form of irresolvable ambiguities, that are prone to generate circular arguments that readily presuppose what they aim to explain. The consequences of this ambiguity are not just theoretical—after all, different metapsychological interpretations tend to suggest different clinical approaches.

## 5 Theoretical and clinical consequences

The distinction between “communicative intention” and “communicative potential” has revealed two different ways to interpret the communicative aspect of projective identification. On the one hand, there are cases where a *spontaneous affective discharge influences the* other, whereas on the other hand, there are cases where one is *deliberately, even if unconsciously, trying to make someone else feel what one feels*. The two interpretations involve differing metapsychological assumptions and commitments, and distinguishing between them seems highly important. The Bionian theory of the communicative aspect of projective identification serves as a reminder to keep track of the less obvious ways in which the patient can connect with the analyst—as many of the already-quoted scholars emphasize, if the analyst manages to remain open and receptive, projective identification can be a powerful tool for understanding the patient’s mind. What I have attempted to underline here is the opposite danger of *reading in* communicative intentions where there might be none, as this is destined to distort our understanding of what is going on in the analytic situation.

In the clinical situation, the distinction between communicative intention and communicative potential might seem *irrelevant* and even disruptive. Much like what Winnicott writes about playing (e.g., Winnicott, 1971, 1–34), the question of whether the presence of the patient *actually harbors* a communicative intention or whether one “only” *acts as if it* does may feel just as inappropriate as the question posed to the caregiver chattering in “motherese” with their newborn infant whether they believe that their baby actually communicates with them or whether they “only” act as if this was the case. However, as should be clear by now, my criticism is not directed at the therapist’s attitude within the clinical situation, nor would I want to claim that parents should not take their infant’s cries as communications. As already indicated, acting *as if* there was a communicative intention in the other’s expression might be something like a *self-fulfilling* prophecy. After all, the caregiver who responds to their child’s cries *as if* they were requests thereby strengthens the child’s sense of the communicative potential of their cry and promotes the emergence of communicative intentions. The same applies, *mutatis* mutandis, to clinical encounters. When the receptive therapist temporarily *immerses* themselves in the affective presence of the patient, *attends* to the affective influence of the latter within themselves *as if* it was an unconscious communication, and spontaneously *responds* to it as such, the therapist’s non-verbal presence (like that of the caregiver) *underline*s the communicative potential of the patient’s spontaneous expressions and thus—in a deferred manner (*nachträglich*)—*invites* the patient to consider their already unfolding spontaneous expressions as communications. That is to say, much like with the sneezing example, the reception *suggests* to the patient—*aprés coup*—that their expressions *were* communications. If the patient plays along, for the time being the communicative quality of the original expression remains unchallenged: by accepting the tacit invitation, so to speak, the patient comes to retrospectively consider their spontaneous expressions as tacit communications.

In this manner, by treating something *as if* it was a communication, it can actually become one. As goes without saying, this can open new ways for the patient to feel connected and understood. What is problematic, however, is when one slides from the attitude of the “as if” into metapsychological statements and treats the clinician’s heuristic insights as psychological facts with direct explanatory force. It is important to keep in mind that the communicative quality was suggested by the patient’s expression only in retrospect (*nachträglich*). The fact that something can be *used as if it was a communication* does not imply that it harbored a communicative intention to begin with. To put it in Freudian terms, even if the analyst can act *as if* the analysand’s entire bodily presence was a case of “chattering with the finger-tips” (Freud, 1905, 78), one should not conclude that the patient *unconsciously aims to* chat something to the analyst. My point here is not that the engaged clinician should distance themselves from their “unchallenged” (Winnicott, 1971, 19) heuristic or playful attitude, constantly reminding themselves that they are “only” acting as if the patient’s presence was a communicative act: this would interrupt their clinical immersion. Instead, my point is that the “magic” of interpersonal encounters should not slide into our *metapsychological assessments* of them. An argumentative slide from “communicative potential” to “communicative intention” can take the form of an explicit theoretical or metapsychological claim, but it can also appear more implicitly in the form of unquestioned metapsychological assumptions and fixed interpretations of what is going on between the patient and the analyst. Both are destined to influence clinical work—sooner or later, and to greater or lesser extent. What we perceive is not unrelated to our theoretical and conceptual understanding. Bluntly put, by stubbornly searching and scanning for phantasies of pushing parts of the self into the other, the analyst is most likely to “discover” such endeavors even where they do not exist. This issue is important because whether an analyst postulates a communicative intention or not thoroughly influences the way they view the patient. To apply the example given earlier of Patient 1 and Patient 2, it is possible that what is actually due to sleep deprivation is seen as being due to psychotic mechanisms within the self, just as it is possible to treat a spontaneous cry as a disguised strategic cry. It is indeed hard to see how this would *not* have a clinical impact.

Moreover, what is crucially different in the two interpretations is not just the *metapsychology of the patient*, but also *the metapsychology of the analyst* (see Fliess, 2007). Specifically, aside from suggesting two remarkably different interpretations of the “pressure exerted” by the patient upon the analyst, and of the patient’s phantasies underlying such activity, the two theories sketch differing pictures of the analyst’s position in relation to the patient. The essential question relates to the one already posed: how can the analyst tell whether there is a communicative intention in the patient or not? The question of whether this is at all possible is perhaps less interesting than the following question: *if the analyst claims to be able to tell this difference, what kind of epistemological position do they thus assign to themselves?*

When one tacitly *believes* or explicitly *claims* that how one feels in the presence of someone else is partly due to that other person’s unconscious attempt to make them feel this way—i.e., when one postulates an unconscious communicative intention in the affective pressure one feels, rather than just acting *as if* there was one–one must accept three daring hypotheses. Namely, to claim knowledge over this, one has to assume that (1) one can, within one’s largely unconscious mental traffic, distinguish *the patient’s contribution from one’s own* (see Spillius, 2012, 53), (2) that one can distinguish *the patient’s unconscious phantasies of the analyst from the patient’s reactions to something actual in the analyst*, and (3) that one can distinguish *countertransferences and reveries that have been intentionally suggested by the patient from those that emerge from the patient’s affective presence without such intentions*. Occasionally, such questions are answered in reference to the analyst’s training and their own analysis. Certainly, people can be better or worse at “mindreading” and in keeping track of their mental traffic, but while psychoanalytic training and the analyst’s own “psycho-analytic purification” (Freud, 1912, 116) are most likely helpful in all this, to assume that these would enable a significant leap toward certitude concerning the mentioned distinctions is nothing but an *idealization*. Indeed, “people do not project into a vacuum—there is always a kernel of reality onto which the projection is hung” (Ogden, 1992, 73; see also Gabbard, 1995, 477), and “not everything a patient feels about his analyst is due to transference, and […] the differentiation between the two kinds of feelings is not always easy” (Heimann 1950, 74). The analyst is not less vulnerable to the tempting illusion of being the master of one’s house, and there is always the danger of treating one’s interpretations “as knowledge rather than as a tentative hypothesis” (Steiner, 2020, 101–102).

In this respect, Klein and Bion differ. While Klein consistently emphasizes the unreliability of the analyst’s subjective experience” (Hinshelwood, 2018, 185), Bion more frequently commits to the aforementioned “slide” both theoretically and clinically. While admitting that “[t]he objection that I project my conflicts and phantasies onto the patient cannot and should not be easily dismissed” (Bion, 1955, 78), Bion notes that *some* of the analyst’s emotional reactions “are dependent on the fact that the analyst […] is in the receiving end of […] projective identification”; and in such cases, according to him, there is “a distinct quality that should enable the analyst to differentiate the occasion when he is the object of a projective identification from the occasion when he is not” (Bion, 1961, 213). Bion’s argument is circular in the same way that Hinshelwood’s is: the presence or absence of projective identification allegedly determines the quality of the analyst’s countertransference, and this qualitative difference is “explained” in reference to whether one is a recipient of projective identification. In all this, the capacity to distinguish between countertransference feelings resulting from the other’s unconscious pressure and those arising otherwise is simply *presupposed*. For a corresponding clinical example, consider the following passage:

“When the patient *strove to rid himself of* fears of death which were felt to be too powerful for his personality to contain *he* split off his fears and *put them into me*, the idea apparently being that if they were allowed to repose there long enough they would undergo modification by my psyche and could then be safely reintrojected” (Bion, 1957a, 103; see also Bion, 1958, 68).

No matter whether this captures what really happened or not (and we have no way of knowing), by *making such a claim* in the first place, Bion *idealizes* his epistemic standpoint as an analyst. Having first noted that the patient wants to *rid* himself of his fear, Bion leaps into saying that the patient has *deliberately pushed* the fear *into him*—thus, in our terms, deducing a communicative intention from a communicative potential. The patient’s fear may well be clearly perceivable in their bodily manner of being, and while immersed in the situation, Bion is forced to feel it too. But whether the fear has been deliberately pushed into him by the patient, Bion really *cannot* know. Instead, he is building on a *hypothesis* and offering *one possible interpretation* of what is happening. Yet, he does not present it this way—he does not say, “I felt like…,” “it was as if…,” or “in the light of the theory of projective identification one could assume that…,” thus underlining that he is making an *interpretation* based on certain theoretical assumptions. Instead, Bion presents his vignette as one describes established facts. In the words of Joseph Sandler: “it is tempting to see all feelings, fantasies, and reactions of the analyst to his patient as being an outcome of what the patient has ‘put into’ the analyst by way of projective identification. Unfortunately, the differentiation of what belongs to the patient and what to the analyst is likely to remain with us for some time as a difficult technical problem” (Sandler, 1987, 26).

If overt cases of psychological manipulation from guilt-tripping to gaslighting (see Hinshelwood, 2018, 101, 197), are brushed aside, it proves practically impossible to tell when the “manipulative element” involves a communicative intention and when it is just simply due to the nature of affects themselves. Insofar as the analyst cannot really know whether the pressure they feel in the presence of the patient harbors a communicative intention or not, it seems appropriate that they should not assume or claim that there is one. As already indicated, by *not assuming a communicative intention*, the analyst is not committed to making *the opposite assumption of the absence of communicative intention*, and in their clinical work, the analyst can remain *neutral* about this, operate in the mode of the *as if*, and thus “assume a less inferential and attributive approach” (Seligman, 2018, 206).^3^ This attitude of not-knowing does not, in other words, prevent the clinician from using the theory of projective identification as a heuristic tool, playfully, in a Winnicottian manner, and seeing where it leads. It is just that when metapsychologically articulating what is going on in the analytic situation, it is all too tempting to consider one’s clinical experiences as unbiased facts and forget that one always has a theoretical and conceptual background that partly prefigures what one seeks—and hence also what one can come to find.

## Conclusion: projective identification, telepathy, and scholarly humility

The fact that affective discharge has a suggestive and pressurizing impact on any preoccupied person present, regardless of whether this impact is intended or not, compels us to reconsider the nature of projective identification and the phantasies underlying it. While the so-called *discharge model* that I have introduced here meets all the criteria for projective identification—in that it includes the phantasy element, the manipulative element, and the reintrojective element—the model does not require the assumption of a *communicative intention* nor the assumption that the underlying phantasy is about moving part of oneself *into the other*. This model is not meant to replace the standard Kleinian–Bionian model. Instead, our previous considerations suggest that projective identification is a broad phenomenon, some cases of which likely involve a communicative intention. However, since one cannot truly know this, one should not slide from attending the felt pressure *as if* it was unconsciously meant as a communication into actually making metapsychological claims about what is happening between the patient and the analyst.

Finally, I want to link these insights to the quote from Freud that we started with—especially the latter half of the quotation that mentions the possibility of preconscious mediation of unconscious communication. The assumption of a communicative intention seems to play a central role in maintaining the psychoanalytic theory of projective identification in its own orbit vis-à-vis the other sciences of non-verbal interaction. By emphasizing our factual ignorance about the presence of such intentions, we open a connection between the psychoanalytic notion and the other sciences of non-verbal interaction.

As mentioned, various sciences have convincingly explored the mechanisms and intricacies of non-verbal communication, and some have even referred to projective identification in doing so (e.g., Schoenewolf, 1990, 58; Gallese, 2009, 531; Arizmendi, 2011, 406). This is not to say that the psychoanalytic notion of projective identification could be altogether *reduced* to what has been discussed in different terms in these other sciences. Yet, I would consider it even more suspicious that other branches of science have nothing to do with what psychoanalysts call projective identification. After all, what we have here is a phenomenon that arguably underpins *all human interaction*—and it would be quite a scandal if the other sciences had missed all of that. In this light, it seems surprising that this extensive research literature is scarcely quoted in psychoanalytic literature on projective identification (see Schafer, 2012, 242). This brings us back to the opening quote from Freud:

“It is a very remarkable thing that the *Ucs* of one human being can react upon that of another, without passing through the *Cs*. This deserves closer investigation, especially with a view to finding out whether preconscious activity can be excluded as playing a part in it; but, descriptively speaking, the fact is incontestable” (Freud, 1915, 193).

The first half of this passage is often cited. The latter half—expressing the possibility that unconscious communication might be mediated by “preconscious activity”—has received much less emphasis. As I see it, the pair of sentences in the quotation nicely captures Freud’s twofold person: on the one hand, there is the unprejudiced creative explorer, while on the other hand, there is the rigorous academic scholar who never ceased to appreciate the ideals of science and remain open to interdisciplinary collaboration (on this, see Whitebook, 2017). In the given passage, it is as if the “first Freud” introduces a revolutionary idea of unconscious communication, and the “second Freud” rushes to add that this might also relate to preconscious experiences—and hence to other sciences dealing with that.

While the first aspect of Freud led him to some of his most innovative discoveries, it also prompted him to examine questions of telepathy, the *descriptions* of which come rather close to those of projective identification. When writing about the former, Freud considers the possibility that “mental processes in one person—ideas, emotional states, conative impulses—can be transferred to another person through empty space without employing the familiar methods of communication by means of words and signs” (Freud, 1933, 39). Indeed, the direct and non-symbolic mode of communication, which is also at issue in projective identification, is prone to invite mystification to which Freud occasionally succumbed. In this regard, the second half of the passage—and indeed the fact that it is quoted so seldom!—is very interesting. Having noted that, *descriptively* speaking, unconscious communication from one person to another is clinically well-documented, Freud adds that “closer investigation” is needed to clarify whether this phenomenon is *enabled and mediated* by “preconscious activity,” and hence by “whatever is implicitly present in mental activity without constituting an object of consciousness” (Laplanche and Pontalis, 1973, 326–327). In light of Freud’s definition of the preconscious, one might assume that these possible *mediating processes* include what we today know as peripheral perception, kinesthetic background awareness, neurophysiological mirroring, embodied simulation, affective contagion, tacit modes of empathy and sympathy—and so on (see Sandler and Joffe, 1967, 219; Seligman, 2018, 196). In effect, psychoanalysis does not seem to be *alone* in addressing the subject matter of unconscious communication—or at least it does not have to be. Yet, for some reason, the suggestion often encounters considerable resistance that other sciences also have something interesting to say about unconscious communication and projective identification. As Schafer puts it, it is as if the inclusion of other sciences would pose a *threat* to psychoanalysis (see Schafer, 2012, 242).

Defensive or not, such exclusion leads to unnecessary mystification. The case of telepathy is quite telling. For when Freud notes that, in what he calls telepathy, something is “transferred” from one person to another “by some unknown method which excluded the means of communication familiar to us” (Freud, 1921, SE 18, 183), he is not saying that such thought-transfer *is not mediated*, but that he *does not know how it is mediated*. To cite another famous passage:

“[the analyst] must turn his own unconscious like a receptive organ towards the transmitting unconscious of the patient. He must adjust himself to the patient as a telephone receiver is adjusted to the transmitting microphone. Just as the receiver converts back into sound waves the electric oscillations in the telephone line which were set up by sound waves, so the doctor’s unconscious is able, from the derivatives of the unconscious which are communicated to him, to reconstruct that unconscious, which has determined the patient’s free associations” (Freud, 1912, 115–116).

The analogy makes it clear that the analysand’s yet-to-be-contained feelings do not mystically reach the analyst without mediation, but in and through “electric oscillations” that we can interpret as metaphors for *preconscious affective interactions*, as suggested in the earlier quotation. To “convert back into sound waves” would, in this reading, refer to how the mind of the analyst (or parent) generates a tentative representation or “trial figuration” based on what they preconsciously receive from the patient (see Sandler and Joffe, 1967, 214). It is understandable that the fact of mediation remains overlooked when focusing on *the content* that is mediated—much like, when absorbed in reading, the line of text with certain color and font “fades out before what is expressed” (Merleau-Ponty, 2002, 466). Yet, even if this mediation is—and cannot be—attended to amid clinical interaction (no more than the text can be attended to as such while reading immersively), this does not justify the claim that it is not there. One approaches such a way of thinking when one refuses to look into the telescope and assumes that the other sciences have nothing to say about the psychoanalytic notion of projective identification. In Sandler’s words, “projective identification is more a descriptive than an explanatory concept” (Sandler, 1987, 26): the *descriptive* undeniability of the phenomenon, to which Freud refers in the opening quotation, is not an explanation of the phenomenon, and to use it as such is to give a “pseudoexplanation” (Sandler, 1987, 26). As Freud writes to Fliess at one point, “I remain loyal to thought-reading and continue to doubt ‘magic’” (see Masson, 1985, 440).

Our choice in this regard is quite straightforward: *either* we make use of the knowledge that we have today about non-verbal communication and risk the possibility that this prompts us to partly revise our habitual metapsychological understanding of what is happening between two persons in the case of projective identification, *or* we refuse to examine this preconscious mediation and withdraw to an isolated and mystifying account of what is going on—perhaps even insisting on using the term “telepathy.” It is not difficult to discern which of these options would be more detrimental to the psychoanalytic tradition.

Although Freud himself does not use the term projective identification, his notes on unconscious communication may thus serve to conclude our argument. The point is quite simple: the communicative aspect of projective identification can be interpreted in two ways—in terms of turning one’s attention to an unconscious *communicative intention* that allegedly was there all along or in terms of *using the communicative potential of the affective presence of the patient*. As the two cases outline a very different metapsychological picture of what is happening between the patient and the therapist, making the distinction is very important. Moreover, given the frequent indistinguishability between these cases, the recipient can avoid idealizing their epistemological position only if they resolutely and comprehensively embrace the state of not-knowing. This resoluteness does not prevent the clinician (or parent) from acting *as if* there was a communicative intention in the other’s presence—problems only emerge when one slides from the heuristic and playful *as if* mode into making metapsychological judgments. That is to say, the analyst does well by determinately occupying a state of “not-knowing”—even if, in their clinical work, they will have to temporarily act *as if* they did know.

## Data Availability

The original contributions presented in the study are included in the article/supplementary material, further inquiries can be directed to the corresponding author.

## Footnotes

1 Given our goal here, we need not go into the related aspects that Klein associates to projective identification – say, its defensive nature, the sense of loss of part of the self, the phantasy of control, and so on. The argument presented here is supposed to apply no matter such features are presupposed or not. I will likewise omit various further distinctions that have been suggested in discussions of projective identification — e.g., Britton’s distinction between “attributive” and “acquisitive” projective identification (Britton, 1998, 6), Spillius’s distinction between “evocatory” and “non-evocatory” projective identification (Spillius, 2012, 58), or Laplanche’s and Pontalis’ distinction between “centrifugal” and "centripetal” projective identification (Laplanche and Pontalis, 1973, 357) — as these carry no effect to the argument presented here.

2 When elaborating further the phenomenology of this “communication,” it might be interesting to make use of J. L. Austin’s speech act theory. Austin famously distinguishes between utterances as “locutionary acts,” as “illocutionary acts,” and as “perlocutionary acts” (Austin, 1962). When uttering, for instance, “there is a storm coming,” as a *locutionary act* the utterance serves as symbolic expression for an expected state of affairs. As an *illocutionary act*, however, the same utterance tacitly hinges upon the possible consequences of the anticipated state of affairs and thus perhaps serves as a warning. As a *perlocutionary act*, moreover, the utterance is way of bringing about an effect in the addressee: speaking of an approaching storm may, for instance, cause fear in the recipient. As goes without saying, rather than being mutually exclusive, these three aspects of an expression can all be simultaneously operative. The mutual emphases between them varies, however. Following this line of thinking, we could assume that if the lastly-mentioned aspect is emphasized enough, the *effect* that the speaker’s expressions have in the recipient may outshine *what* the speaker is literally referring to or *what* they are implying. This could perhaps also be developed vis-à-vis non-verbal communication as well, even if Austin does not do this. Developing such connections in detail would require another paper, however. I want to thank my Reviewers for bringing this interesting link to my attention.

3 In this connection, one could also discuss the difference between “interpretative interventions” and “affirmative interventions” (Killingmo, 1989, 74), between “functional,” “complementary” and “empathic” responses of the analyst (Tähkä, 1994, 13–90), and what Ogden has called “epistemological” and “ontological” psychoanalysis (Ogden, 2022b, 10–15).
